# Participatory mapping identifies risk areas and environmental predictors of endemic anthrax in rural Africa

**DOI:** 10.1038/s41598-022-14081-5

**Published:** 2022-06-22

**Authors:** Olubunmi R. Aminu, Taya L. Forde, Divine Ekwem, Paul Johnson, Luca Nelli, Blandina T. Mmbaga, Deogratius Mshanga, Mike Shand, Gabriel Shirima, Markus Walsh, Ruth N. Zadoks, Roman Biek, Tiziana Lembo

**Affiliations:** 1 Institute of Biodiversity, Animal Health & Comparative Medicine, University of Glasgow, Glasgow, UK; 2 Nelson Mandela African Institution of Science and Technology, Arusha, Tanzania; 3 Kilimanjaro Christian Medical University College, Moshi, Tanzania; 4 Kilimanjaro Clinical Research Institute-Kilimanjaro Christian Medical Centre, Moshi, Tanzania; 5 Tanzania Veterinary Laboratory Agency, Northern Zone, Arusha, Tanzania; 6 School of Geographical & Earth Sciences, University of Glasgow, Glasgow, UK; 7 International Center for Tropical Agriculture, Selian Agricultural Research Institute, Arusha, Tanzania; 8 Sydney School of Veterinary Science, University of Sydney, Sydney, Australia

**Keywords:** Infectious diseases, Biogeography, Microbial ecology

## Abstract

Disease mapping reveals geographical variability in incidence, which can help to prioritise control efforts. However, in areas where this is most needed, resources to generate the required data are often lacking. Participatory mapping, which makes use of indigenous knowledge, is a potential approach to identify risk areas for endemic diseases in low- and middle-income countries. Here we combine this method with Geographical Information System-based analyses of environmental variables as a novel approach to study endemic anthrax, caused by the spore-forming bacterium *Bacillus anthracis*, in rural Africa. Our aims were to: (1) identify high-risk anthrax areas using community knowledge; (2) enhance our understanding of the environmental characteristics associated with these areas; and (3) make spatial predictions of anthrax risk. Community members from the Ngorongoro Conservation Area (NCA), northern Tanzania, where anthrax is highly prevalent in both animals and humans, were asked to draw areas they perceived to pose anthrax risks to their livestock on geo-referenced maps. After digitisation, random points were generated within and outside the defined areas to represent high- and low-risk areas, respectively. Regression analyses were used to identify environmental variables that may predict anthrax risk. Results were combined to predict how the probability of being a high-risk area for anthrax varies across space. Participatory mapping identified fourteen discrete high-risk areas ranging from 0.2 to 212.9 km^2^ in size and occupying 8.4% of the NCA. Areas that pose a high risk of anthrax were positively associated with factors that increase contact with *Bacillus anthracis* spores rather than those associated with the pathogen’s survival: close proximity to inland water bodies, where wildlife and livestock congregate, and low organic carbon content, which may indicate an increased likelihood of animals grazing close to soil surface and ingesting spores. Predicted high-risk areas were located in the centre of the NCA, which is likely to be encountered by most herds during movements in search for resources. We demonstrate that participatory mapping combined with spatial analyses can provide novel insights into the geography of disease risk. This approach can be used to prioritise areas for control in low-resource settings, especially for diseases with environmental transmission.

## Introduction

Understanding the spatial distribution of infectious diseases and the factors driving geographical heterogeneities in their incidence is fundamental to epidemiology^1^. However, in many low- and middle-income countries where disease burdens are high, our ability to map diseases and unravel their underlying processes is compromised by limited infrastructure and resources for surveillance. This is especially the case in the most remote areas where systems for disease surveillance are typically lacking and are beyond the means of local health budgets. Economic constraints also adversely affect the ability of local governments to implement appropriate disease prevention and control strategies to respond to disease threats. To enable prioritisation of areas for targeted interventions, low-cost approaches for mapping areas of disease occurrence and risk are therefore required.

A number of methods may be employed to map spatial patterns of disease, including those that utilise incidence data from passive surveillance, active surveillance and surveys^2–6^. However, these methods may provide an incomplete picture (e.g. passive surveillance) or may be impractical and costly to implement in remote areas with limited infrastructure (e.g. active surveillance and targeted surveys)^7^. In addition, depending on the nature of the pathogen under investigation, sampling for disease confirmation may pose health risks to operators on the ground. An alternative approach that offers promise is the use of participatory mapping. This is a form of participatory research, reliant on the generation of research data through consultations with relevant local communities, thereby making use of indigenous knowledge^8^. For example, pastoralists of East Africa are renowned for their understanding of their environment, and their ability to recall incidents relating to their livestock’s health and productivity even over long periods of time^9,10^. Participatory mapping tools are increasingly being used for disease surveillance; successful examples include applications to vector-borne disease control such as mapping mosquito abundance to understand risk areas for dengue^11^ or malaria^12^. Moreover, where these approaches can be integrated with Geographic Information System (GIS) and spatial analyses, they have strong potential to generate more quantitative and mechanistic information about disease risk^13^.

One pathogen that typifies many of the surveillance challenges discussed above in low- resource settings is *Bacillus anthracis*. *B. anthracis* is a spore-forming bacterium that causes anthrax, a zoonotic disease (i.e. a disease that can be transmitted between animals and humans) that remains endemic in many developing countries. Anthrax leads to sudden death in herbivorous animals (both livestock and wildlife) and is potentially fatal in humans who contract infection from handling or eating carcasses, animal products or biological materials contaminated with *B. anthracis* spores. Once spores enter the host, they germinate into vegetative forms capable of replicating and producing toxins that are the eventual cause of death^14^. Upon death of the host, the vegetative forms are released into the environment where they sporulate, hence perpetuating the cycle. Anthrax spores may persist for decades in contaminated soil^14^, presenting a long-lasting risk of infection to susceptible hosts.

Anthrax endemic areas are often located in remote settings which makes surveillance problematic. Therefore, data on human anthrax cases tend to be patchy and insufficient to reflect distribution of disease. Case under-reporting and under-/mis-diagnosis are important reasons for the limited data available. For example, affected individuals are often unable to access medical facilities due to poor infrastructure (e.g. roads) or the high costs involved^15^, and therefore medical records poorly reflect disease incidence. Limited awareness of the clinical presentation of the disease may also be responsible for poor reporting. This is especially the case for the forms of the disease characterised by less specific clinical signs and symptoms, or by diagnostic challenges, for example gastrointestinal anthrax^16^. Our earlier studies in Tanzania highlighted that existing medical records are insufficient to reflect disease incidence, but that case-fatality rates are as high as 50% when cases are actively investigated^4^. Official data on anthrax in animals are equally patchy. An additional challenge to anthrax surveillance is that its detection involves the collection of highly infectious diagnostic samples from animal or human hosts or the environment. The resulting biosafety hazards lead to reluctance to engage in field surveillance, and therefore a general lack of data on fine-scale disease patterns. In addition, in most anthrax-endemic areas, animal carcass survival is challenged by high ambient temperatures, and presence and activity of local scavenger populations. Even when samples are collected, poor diagnostic expertise and infrastructure limit their use for diagnostic confirmation^17^. The spatial risk of anthrax has never been studied using participatory mapping methods, yet much can be gained from local communities’ knowledge of the disease and of the environments that may pose the greatest risks to their animals. To address this gap, our study focuses on anthrax in hyper-endemic settings of northern Tanzania, to identify and characterize areas of risk using participatory mapping as a safe alternative to traditional surveillance.

For pathogens with an environmental phase, like *B. anthracis,* information on areas of risk as determined through participatory mapping can be combined with environmental data to understand factors that make these areas suitable for pathogen persistence. Factors associated with the survival of *B. anthracis* in the environment include temperature, pH, nutrient and oxygen availability, all thought to also influence sporulation^18^, as well as precipitation and moisture, organic matter, inorganic calcium, vegetation and soil topography^19^. Since anthrax occurrence is often localised, geographical areas affected by the disease may be characterised by distinct environmental features, but these heterogeneities are not well understood^2^. Factors that promote the occurrence of anthrax can be grouped into conditions that are associated with the survival of *B. anthracis* in the environment (spore survival) and/or the likelihood of spores coming in contact with a suitable host (exposure). For instance, soil alkalinity and both extreme drought and rainfall have been associated with anthrax occurrence in some parts of Africa^20–22^. While dry conditions (e.g. caused by lack of rainfall) and alkaline soil promote spore formation and survival, heavy rainfall and close proximity to water bodies may facilitate transmission to animals by the action of water unearthing spores and washing them into water bodies where animals congregate. Arguably, spore survival and transmission are both critical, however it is unclear whether both drive the risk of anthrax to the same extent.

A participatory mapping approach is particularly well suited to the study of anthrax in pastoral communities, who typically move their livestock in search of resources (e.g. grazing and watering). Since the incubation period for animal anthrax can range from a few hours to 21 days^14^, an infected animal may travel a long distance before clinical signs appear or death occurs. This means that environmental characteristics of the place where an animal became infected might differ from those where it succumbed to the disease. However, livestock owners will know where their animals had been at the likely time of infection and thus develop knowledge about potential risk areas over time. Participatory mapping allows us to tap into this knowledge and thus to acquire epidemiologically relevant information that is not available from mapping disease cases based on the location of occurrence.

In this study, we mapped animal anthrax-risk areas as defined by livestock-owning communities during participatory mapping sessions in an anthrax-endemic region of Tanzania, the Ngorongoro Conservation Area (NCA). We subsequently tested whether areas of risk were linked with environmental factors that had been previously shown to be associated with anthrax occurrence. These analyses enabled us to enhance our understanding of environmental characteristics that might explain why perceived risk areas are likely to be favourable for anthrax occurrence, in terms of spore survival and/or transmission to animals. We used this information to predict the risk of anthrax across the NCA.

## Methods

### Study area

**Figure 1:**
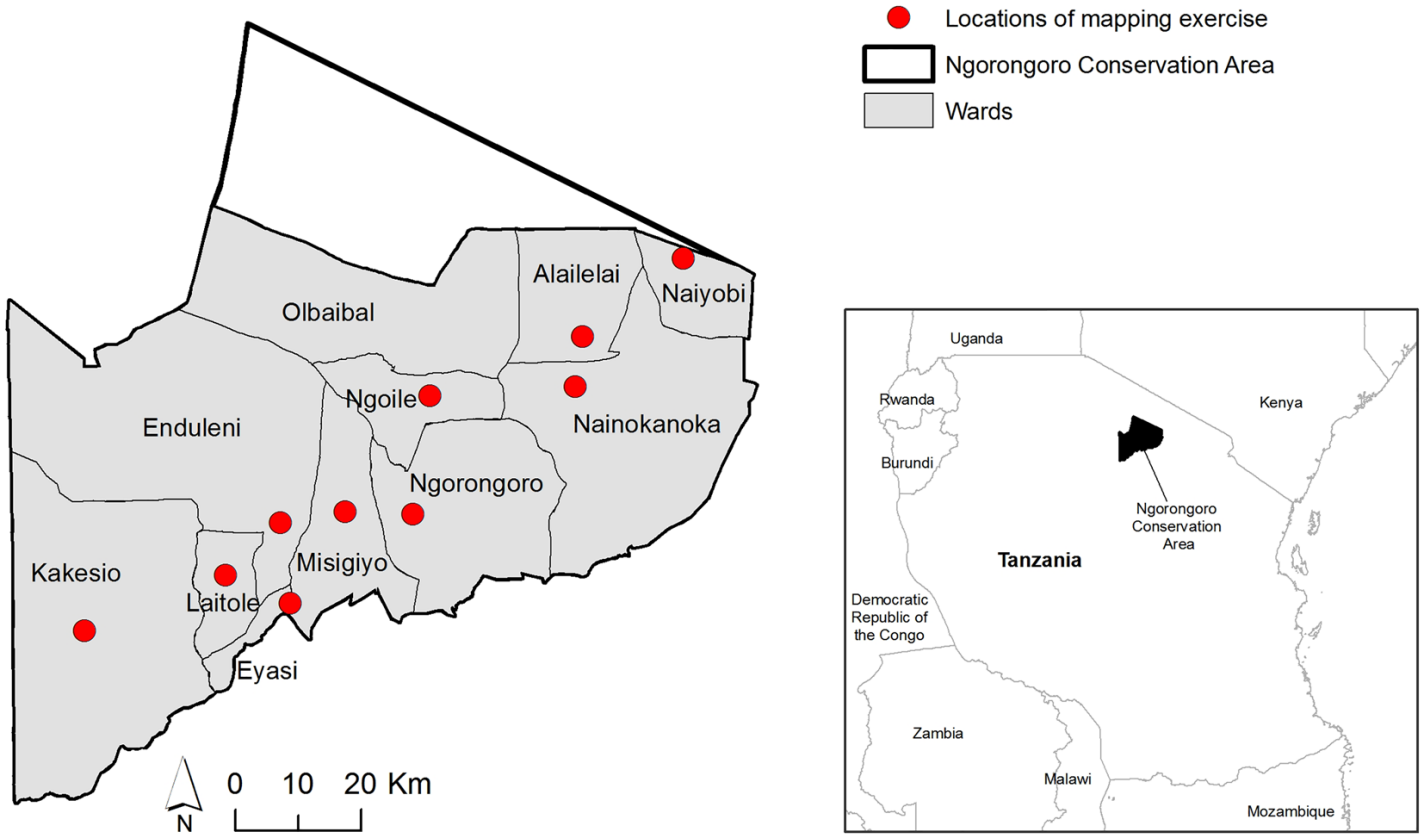
Locations of participatory mapping. Map showing the 11 administrative wards of the Ngorongoro Conservation Area in northern Tanzania and the locations where participatory mapping sessions took place (red dots). The maps were produced in QGIS 2.18.2 using data from the National Bureau of Statistics, Tanzania (http://www.nbs.go.tz/).

The NCA encompasses an area of 8292 km^2^ and in 2020 had approximately 87,000 inhabitants^23^, who are primarily dependent on livestock for their livelihoods. It is a multiple-use area where people coexist with wildlife and livestock, and practise pastoralism with transhumance, characterised by seasonal movements of livestock for accessing resources such as grazing areas and water. The NCA comprises eleven administrative wards: Alailelai, Endulen, Eyasi, Laitole, Kakesio, Misigiyo, Ngorongoro, Naiyobi, Nainokanoka, Ngoile and Olbalbal (Fig. 1). The NCA was chosen for our study as it is known to be hyperendemic for anthrax^4,17,20^. In addition, informal consultations we held prior to the study, as well as tailored data collection at the community and household level, indicated that local communities have a good understanding of the disease in humans and animals, and of practices around carcass and livestock management that increase risks, particularly in certain locations and periods of the year^24^.

### Ethics approval and consent to participate

The study received approval from the National Institute for Medical Research, Tanzania, with reference number NIMRJHQ/R.8a/Vol. IX/2660; the Tanzania Commission for Science and Technology (numbers 2016-94-NA-2016-88 (O. R. Aminu), 2016-95-NA-2016-45 (T. L. Forde) and 2018-377-NA-2016-45 (T. Lembo)); Kilimanjaro Christian Medical University College Ethics Review Committee (certificate No. 2050); and the University of Glasgow College of Medical Veterinary & Life Sciences Ethics Committee (application number 200150152). Approval and permission to access communities and participants were also obtained from relevant local authorities. Written informed consent was obtained from all participants involved in the study. All data collected were analysed anonymously, ensuring the confidentiality of participants. All research activities were performed in accordance with relevant guidelines and regulations.

### Participatory mapping

A participatory mapping approach based on methodology previously tested in East Africa^25^ was employed to define areas of anthrax risk for animals in the NCA based on community knowledge. Georeferenced maps of the NCA were produced using data from Google and DigitalGlobe (2016). The maps used datum Arc 1960/UTM zone 36S and grid intervals of 1000 km and were produced at 1:10,000 and 1:50,000 scales, in order to provide participants with a choice. Ten participatory mapping focus groups were held at ward administrative level (Fig. 1) in order to identify areas in the NCA that communities perceive as posing a high risk of anthrax. One mapping exercise was held in each ward. Ngoile and Olbalbal wards were covered at the same time and treated as one, as they had only recently (in 2015) been split from one ward (Olbalbal). Each session had between ten and thirteen participants, who consisted of village and ward administrators, animal health professionals (including community animal health workers and livestock field officers), community leaders, and selected community members. These participants represented members of the community concerned with animal health and owning livestock and, as such, were likely to hold in-depth knowledge relating to community experience of animal health and disease, including anthrax. Participants were recruited by consulting with animal health professionals as well as village and ward administrators, who gave permission to conduct the mapping sessions.

**Figure 2:**
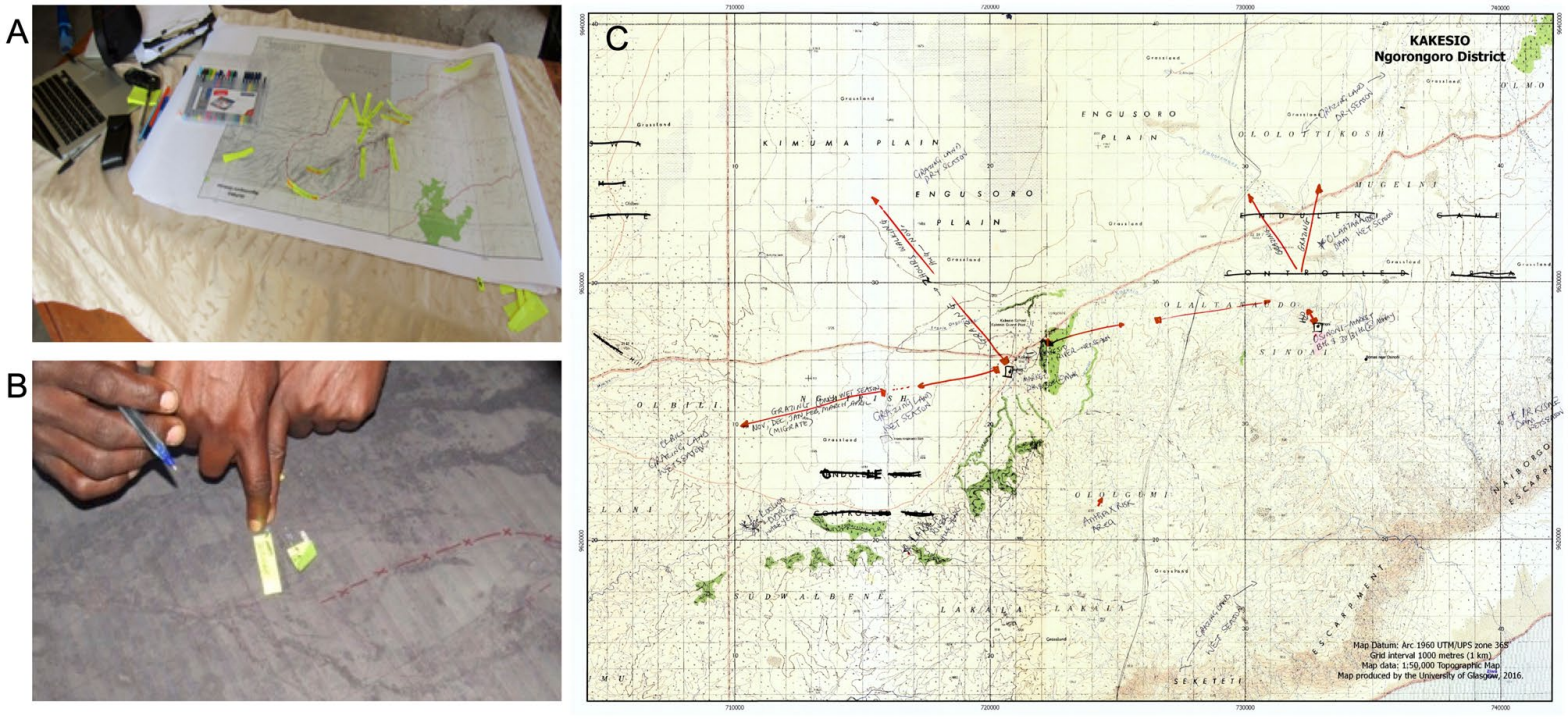
Participatory mapping of anthrax risk areas in the Ngorongoro Conservation Area. Images show (**A**) the set-up of a mapping session, (**B**) participants engaged during a session and (**C**) an example of a 1:50,000 scale map annotated by participants. The map was created with QGIS opensource mapping software. The basemap used was a scanned and geo-referenced full colour 1:50,000 scale topographic map produced by the Surveys & Mapping Division, Ministry of Lands, Housing & Human Settlements, Dar es Salaam, Tanzania. The grid is based on the Arc1960 UTM 36S projection and datum. The map was exported from QGIS in Acrobat Pdf format to enable it to be printed at suitable sizes for using in the fieldwork and to be manually annotated during the participatory mapping.

The mapping sessions were conducted in Swahili and translated into English by an interpreter. Participants’ general knowledge of the area was first verified by testing whether they could correctly identify popular locations such as health centres, places of worship, markets and schools. Subsequently, participants discussed among themselves and came to a consensus about areas they considered to be at high risk of anthrax. Specifically, we asked them to identify locations they perceived as areas where they considered their animals to be at risk of being exposed to anthrax. These areas were drawn on the maps provided (Fig. 2). While they did not locate areas where the animals had succumbed to disease, we also asked for generic information on locations where anthrax outbreaks had occurred in the past to define areas that could be targeted for active surveillance of cases. In order to improve the fidelity of the data, participants defined risk areas in relation to their own locality (ward) and locations where their animals access resources. Therefore, the areas were not defined by administrative boundaries, as communities may access locations outside their wards, for instance for grazing or watering. The resulting maps were scanned, digitised and analysed as detailed in the following sections. Further detail on the participatory mapping process is provided in the Supplementary Methods (Additional File 1).

### Digitisation of maps and generation of random points

Scanned maps were saved as PDF files and converted to high resolution TIFF files for digitisation in QGIS 2.18.2-Las Palmas free OpenSource software^26^. All maps were georeferenced with geographical coordinates during production and reference points were available to enable the precise mapping of all locations. The digitization was carried out using the QGIS digitizing tools and by creating polygon layers of the defined risk areas.

### Sourcing data on the environmental predictors of anthrax

Available soil and environmental data (250 m grid) for Tanzania were obtained from various sources (Table 1). From the available data, we selected the following seven variables which have previously been shown to contribute to or explain the risk of anthrax based on the biology of *B. anthracis* (Table 1).

**Table 1:** Environmental factors with potential to influence anthrax occurrence.

Variable	Source (website)	Research evidence associated with variable	Predicted association with high-risk areas
Cation exchange capacity (CEC)	SoilGrids (https://soilgrids.org)	^19,22^	Positive. CEC is used as a proxy for soil calcium. High calcium promotes anthrax persistence
pH	SoilGrids (https://soilgrids.org)	^19^	Positive. *B. anthracis* spores have been shown to survive best in alkaline soils (pH > 6)
Distance to inland water bodies (DOWS)	SurfaceWater (https://global-surface-water.appspot.com/)	^18,20,22^	Unknown. Anthrax has been shown to occur in dry areas but has also been reported to occur near water sources
Average enhanced vegetation index (EVI), 2000–2016	Africa Soil Information Service (http://africasoils.net/)	^19,20^	Unknown. Higher or lower EVI may promote the risk of anthrax
Average daytime land surface temperature (LSTD), 2001–2015	Africa Soil Information Service (http://africasoils.net/)	^19^	Positive. Anthrax occurrence is associated with places having elevated temperature
Slope	Open Topography (http://opentopo.sdsc.edu)	^19^	Negative. Anthrax has been observed more often in flat topography
Predicted topsoil organic carbon content (SOC)	SoilGrids (https://soilgrids.org)	^18^	Positive. Soils with high organic matter may retain spores more readily

Data were obtained for Tanzania in 2017.

#### Cation exchange capacity (CEC)

Measured in cmol/kg, CEC is the total capacity of the soil to retain exchangeable cations such as Ca^2+^, Mg^2+^ etc. It is an inherent soil characteristic and is difficult to alter significantly. It influences the soil's ability to hold on to essential nutrients and provides a buffer against soil acidification^27^. CEC has been reported to be positively correlated with anthrax risk. In addition, CEC is a proxy for calcium content, which may contribute to anthrax risk in a pH-dependent manner as explained below^19,22^.

#### Predicted topsoil pH (pH)

Soil pH below 6.0 (acidic soil) is thought to inhibit the viability of spores^19^ thus a positive effect of higher pH on the risk of anthrax is expected. It has been suggested that the exosporium of *B. anthracis* is negatively charged in soils with neutral to slightly alkaline pH. This negative charge attracts positively charged cations in soil, mainly calcium, enabling the spores to be firmly attached to soil particles and calcium to be maintained within the spore core, thereby promoting the viability of *B. anthracis*^19,28^.

#### Distance to inland water bodies (DOWS)

Both the distance from water and proximity to water may increase anthrax risk. Distance to inland water may indicate the degree to which an area is dry/arid. Anthrax outbreaks have been shown to occur in areas with very dry conditions^19^. Although anthrax occurrence has also been associated with high soil moisture, this relates more to the spore germination in the environment (a mechanism that is disputed) and the concentration of spores in moist humus that amount to an infectious dose^18,29^. Spores will survive much longer in soils with low moisture content^19^. Low moisture may also be associated with low vegetation which results in animals grazing close to the soil, increasing the risk of ingesting soil with spores. Hampson et al*.* reported that anthrax outbreaks occurred close to water sources in the Serengeti ecosystem of Tanzania in periods of heavy rainfall^20^, and Steenkamp et al. found that close proximity to water bodies was key to the transmission of *B. anthracis* spores in Kruger National Park, South Africa^22^. Water is an important resource for livestock and a large number of animals may congregate at water sources during dry seasons. The close proximity of a water source to a risk area may increase the chance of infection, particularly during periods of high precipitation which might unearth buried spores.

#### Average enhanced vegetation index (EVI)

Vegetation density may influence the likelihood of an animal ingesting soil or inhaling dust that may be contaminated with spores. Grazing animals are more likely to encounter bacteria in soil with low vegetation density^20^, although there is a possibility that spores can be washed onto higher vegetation by the action of water^19^. Vegetation index may also reflect the moisture content of soil. Arid/dry conditions favour the formation and resistance of spores in the environment, thus lower vegetation may be associated with the occurrence of anthrax.

#### Average daytime land surface temperature (LSTD)

Anthrax has been more commonly reported to occur in regions with warmer climates worldwide. Minett observed that under generally favourable conditions and at 32 °C to 37 °C, sporulation of *B. anthracis* occurs readily but vegetative cells are more likely to disintegrate at temperatures below 21 °C^30^. Another hypothesis for the association of high temperature with anthrax occurrence is altered host immune response to disease due to stress caused by elevated temperatures^19^. In addition, elevated temperatures are usually associated with arid areas where vegetation is low, limiting access to adequate nutrition, which in turn affects immunity. Similarly, in hotter climates where infectious diseases occur more often, host interactions with other pathogens may modulate immune response to anthrax^31^. In this case, a lower infectious and lethal dose of spores would be sufficient to cause infection and death, respectively^19^. Contact with and ingestion of soil, spores and abrasive pasture is also higher with low vegetation in hot and arid areas^19,32^. In boreal regions such as in northern Canada, where anthrax occurs in wood bison, and Siberia, the disease is more commonly reported in the summer^19^. We therefore hypothesised a positive effect of LSTD on the risk of anthrax.

#### Slope

Spores of *B. anthracis* are hypothesized to persist more easily in flat landscapes that are characterised by shallow slopes^19^, as it is thought that wind and water may disperse spores more easily along areas with a higher slope gradient, thereby decreasing the density of spores to levels that may be insufficient to cause infection in a susceptible host. Therefore, we expected a negative relationship between slope and the risk of anthrax.

#### Predicted topsoil organic carbon content (SOC)

Organic matter (g/kg) may aid spore persistence by providing mechanical support. The negatively charged exosporium of spores is attracted to the positive charges on hummus-rich soil, thus anthrax is thought to persist in soil rich in organic matter^18^. Based on available evidence, we expected a positive effect of SOC on the risk of anthrax.

### Creating the dataset

**Figure 3:**
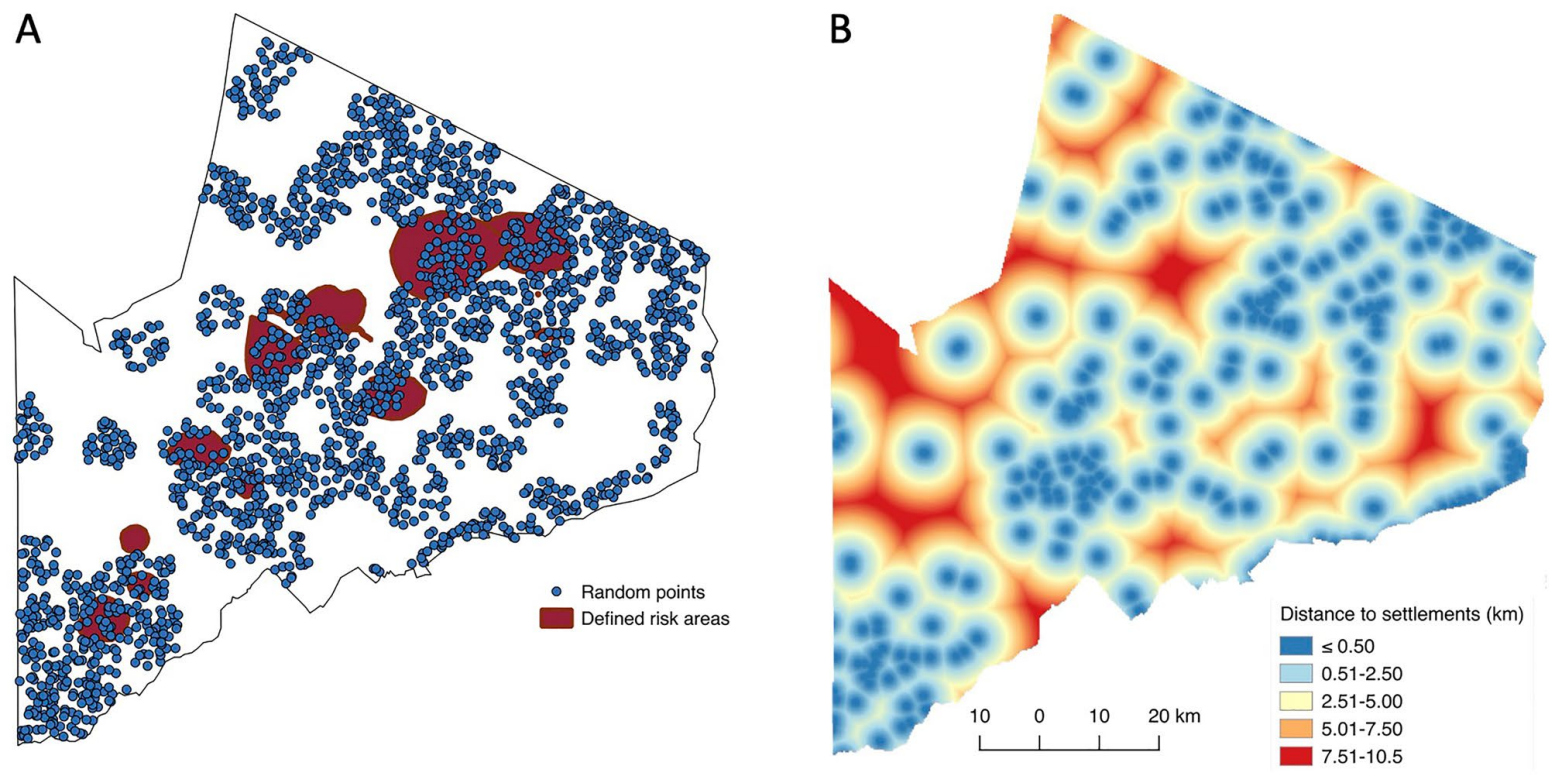
Ngorongoro Conservation Area map showing (**A**) defined risk areas (in red) and (**B**) distance to settlements. For analysis, 5000 random points were generated throughout the area; points falling within 4.26 km of human settlements (the average distance herds are moved from settlements in a day as determined through interviews of resident livestock owners) were retained for analysis (n = 2173, shown in blue in 3a). The maps were created in QGIS 2.18.2 using data from the National Bureau of Statistics, Tanzania (http://www.nbs.go.tz/).

The annotated and digitised maps yielded polygons of high-risk areas within the NCA (Fig. 3). After digitization, 5000 random points were generated^33^ to cover the 8292 km^2^ area of the NCA. This enabled us to obtain distinct points allowed by the 250 m grid resolution of the environmental variables. Points falling within the defined risk areas were selected to represent risk areas while those falling outside represented low-risk areas. Measures of the environmental characteristics associated with individual points were obtained with the ‘add Raster data to points’ feature in QGIS.

In order to focus on areas of greatest risk to humans and livestock and to exclude locations that are not accessible, only points within a certain range of distance from settlements were included (Fig. 3). On average, herders in the NCA move their livestock 4.26 km away from settlements for grazing and watering during the day (unpublished data obtained through a cross-sectional survey of 209 households). Thus, only points falling within this distance from settlements were selected, providing us with data on areas where infection is most likely to occur. Data on locations of settlements were obtained from satellite imagery and included permanent residences as well as temporary settlements (e.g. seasonal camps set up after long distance movement away from permanent settlements, typically in the dry season, in search of pasture and water). These data were collated from the Center for International Earth Science Information Network (CIESIN).

After adjusting for accessibility of resource locations using the average distance moved by livestock, 2173 points were retained for analysis, of which 239 (11%) fell within high-risk areas.

### Data analysis

All statistical analyses were carried out in R (v 4.1.0) within the RStudio environment^34^. The aims of the statistical analysis were to infer the relationship between anthrax risk areas as determined through participatory mapping and the environmental factors identified in Table 1, and to use this relationship to make spatial predictions of anthrax risk across the study area. We achieved both aims by modelling the binary risk status (high or low) of the randomly generated points as a function of their environmental characteristics in a Bayesian spatial logit-binomial generalised linear mixed-effects model (GLMM), implemented in the package *glmmfields*^35^. Spatial autocorrelation (residual non-independence between nearby points) was accounted for by including spatial random effects in the GLMM. We chose relatively non-informative priors for the intercept and the covariates, using Student’s t-distributions centred at 0 and wide variances (intercept: df = 3, location = 0, scale = 10; betas: df = 3, location = 0, scale = 3). For the spatial Gaussian Process and the observation process scale parameters, we adopted the default *glmmfields* settings and used half-t priors (both gp_theta and gp_sigma: df = 3, location = 0, scale = 5), and 12 knots. To achieve convergence, the models were run for 5000 iterations^35^.

First, univariable models were fitted to estimate unadjusted associations between each environmental factor (CEC, pH, DOWS, EVI, LSTD, slope, and SOC; Table 1; Supplementary Table S1) and high- and low-risk areas. Second, we constructed multivariable models by fitting multiple environmental variables (Supplementary Table S2). Three variables, SOC, slope and EVI showed a strongly right-skewed distribution and were therefore log-transformed prior to GLMM analysis to prevent excessive influence of outliers. All predictor variables were centred to zero mean and scaled to unit standard deviation for analysis, and odds ratios were rescaled back to the original units for ease of interpretation. Prior to fitting the multivariable GLMM, the presence of collinearity among the predictor variables—which were all continuous—was assessed using variance inflation factors (VIFs)^36^, calculated with the *car* package and illustrated using scatter plots (Supplementary Fig. S1)^36^. Three predictor variables showed a VIF greater than 3 (LSTD, ln EVI and pH with VIFs of 6.8, 4.2 and 3.5, respectively). Removal of LSTD and ln EVI reduced all VIFs to below 3, therefore these two variables were excluded from the multivariable regression analysis^37^.

The model performance was assessed by calculating the area under the receiver operating characteristic curve. The predicted probability of being an anthrax high-risk area was determined and depicted on a map of the NCA using a regular grid of points generated throughout the NCA with one point sampled every 500 m.

### Consent for publication

Permission to publish was granted by the National Institute for Medical Research, Tanzania.

## Results

**Figure 4:**
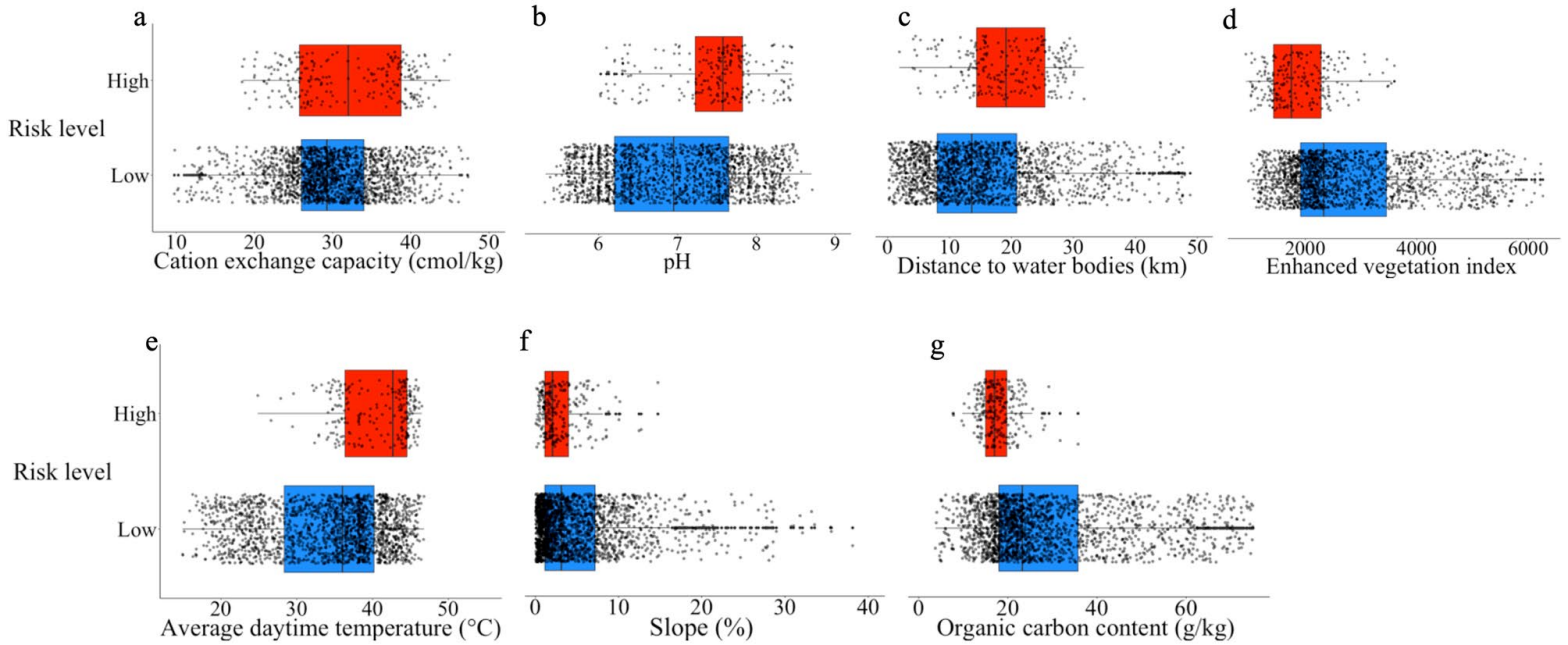
Boxplots of environmental variables. Data values, median, lower and upper quartiles are shown for points falling in both anthrax high- (red) and low-risk (blue) areas as defined through participatory mapping in the Ngorongoro Conservation Area. Plots are shown for (**a**) cation exchange capacity, (**b**) pH, (**c**) distance to inland water bodies, (**d**) average enhanced vegetation index, (**e**) average daytime land surface temperature, (**f**) slope and (**g**) predicted topsoil organic carbon content. This visual exploration does not account for spatial autocorrelation of points.

**Figure 5:**
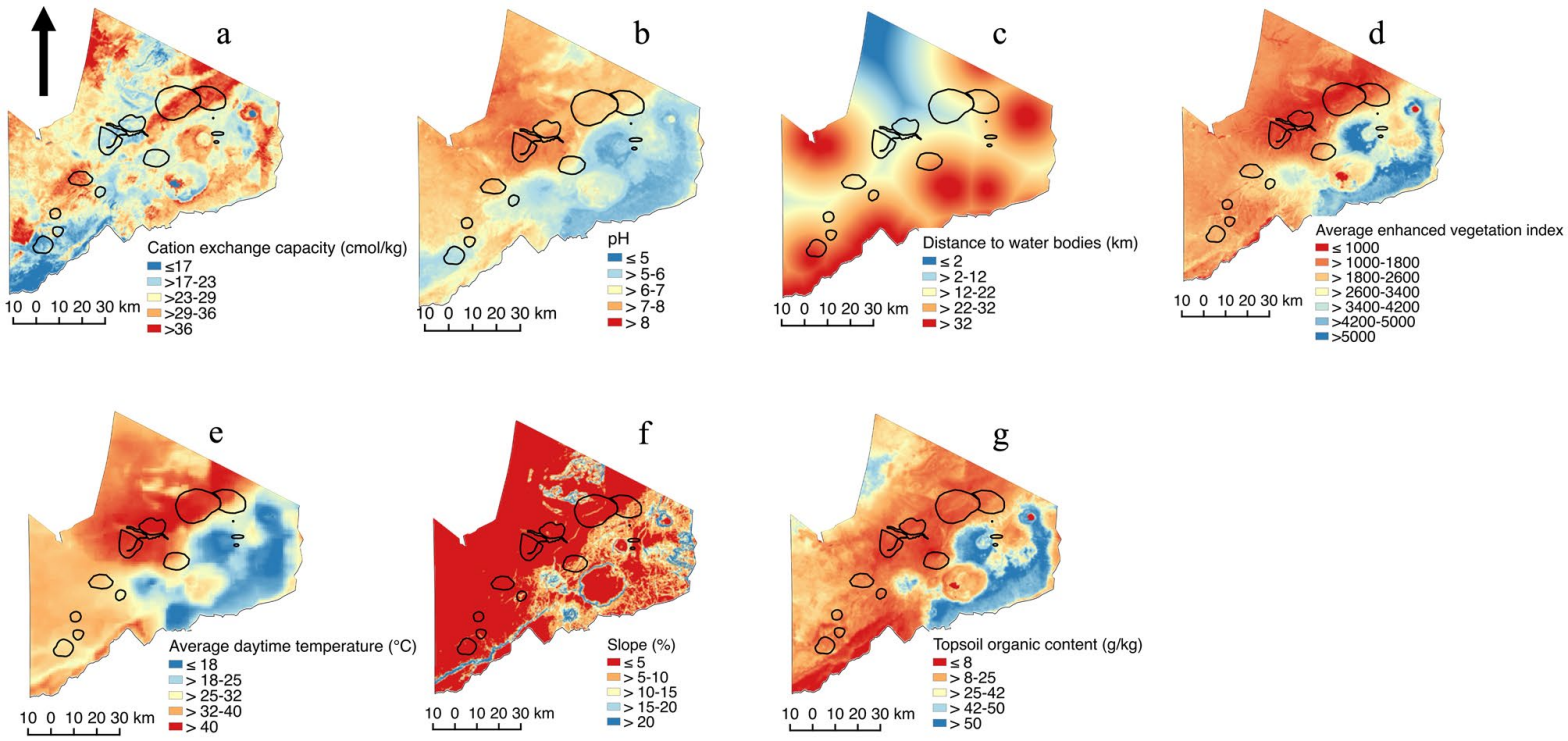
Hypothetical environmental predictors of anthrax in the Ngorongoro Conservation Area, Tanzania. (**a**) cation exchange capacity, (**b**) pH, (**c**) distance to water bodies, (**d**) enhanced vegetation index, (**e**) daytime temperature, (**f**) slope and (**g**) topsoil organic carbon content. Based on available scientific evidence (Table 1), areas with warm colours (red, orange and yellow) have environmental conditions favourable to a high risk of anthrax, while areas with cool colours (blue) have conditions associated with a low risk. Perceived risk areas identified using participatory mapping exercises with indigenous communities are enclosed by black lines. The maps were created in QGIS 2.18.2 using data obtained from the Center for International Earth Science Information Network (CIESIN). Maps of the Ngorongoro Conservation Area were sourced from the National Bureau of Statistics, Tanzania (http://www.nbs.go.tz/).

Fourteen discrete high-risk areas ranging from 0.2 to 212.9 km^2^ in size and with a total area of 695.3 km^2^ were identified through the participatory mapping sessions. These high-risk areas occupy 8.4% of the total area in the NCA. The environmental covariates are described and depicted in Table 2, Figs. 4 and 5.

**Table 2:** Descriptive characteristics of the environmental covariates used to investigate the risk of anthrax in the Ngorongoro Conservation Area.

Variable	Range	Median (interquartile range) in high-risk areas	Median (interquartile range) in low-risk areas
Cation exchange capacity (CEC) cmol/kg	9.75–47.25	32.00 (13.00)	29.30 (8.00)
pH	5.32–8.70	7.58 (0.60)	6.95 (1.45)
Distance to inland water bodies (DOWS) km	0.25–48.84	19.10 (11.04)	13.58 (12.85)
Average enhanced vegetation index (EVI)	934.00–6261.00	1750.00 (851.00)	2330.00 (1541.50)
Average daytime land surface temperature (LSTD) °C	14.94–46.79	42.67 (8.18)	36.02 (11.85)
Slope %	0.04–38.12	2.06 (2.85)	3.12 (6.04)
Predicted topsoil organic carbon content (SOC) g/kg	3.75–88.00	17.00 (4.75)	23.50 (18.75)

The univariable GLMM analysis indicated that high-risk areas were closer to permanent water bodies (DOWS), had higher pH, lower organic carbon content (SOC), lower vegetation density (EVI) and higher daytime temperatures (LSTD) (Table 3). There was no evidence that cation exchange capacity (CEC) or slope were associated with the high-risk areas. The result observed for DOWS after taking spatial autocorrelation into account was inconsistent with descriptive data analysis where the mean DOWS was higher for points falling in high-risk areas.

**Table 3:** The odds ratio of points falling into defined risk areas as explained by environmental variables.

Variable	Odds ratio for univariable analysis (95% credible interval)	Odds ratio for multivariable analysis (95% credible interval)
Cation exchange capacity (CEC) cmol/kg	0.980 (0.932–1.002)	1.002 (0.953–1.053)
pH	2.51 (1.336–4.572)*	1.695 (0.806–3.528)
Distance to inland water bodies (DOWS) km	0.878 (0.810–0.951)*	0.907 (0.833–0.987)*
Average enhanced vegetation index (EVI)	0.963 (0.950–0.975)*	–
Average day time land surface temperature (LSTD) °C	1.093 (1.059–1.130)*	–
Slope %	1.002 (0.999–1.004)	1.003 (1.001–1.006)
Predicted topsoil organic carbon content (SOC) g/kg	0.973 (0.963–0.982)*	0.975 (0.963–0.986)*

Results of univariable and multivariable analysis showing the association between the probability of high anthrax risk and environmental variables in the Ngorongoro Conservation Area. EVI and LSTD were excluded from the multivariable model due to a high degree of collinearity as indicated by a variance inflation factor > 3. (*) gives indication of an association (i.e. credible intervals excluding 1).

The multivariable GLMM showed that the probability of points falling in high-risk areas did not depend on pH or CEC (Table 3). However, proximity to water bodies (DOWS), and low organic carbon content (SOC) were clearly associated with high-risk areas (odds ratios 0.907 and 0.975, respectively; Table 3). Using the area under the receiver operating characteristic curve as a metric for assessing within-sample predictive performance of the multivariable model, the area under the curve (AUC) of 0.94 obtained indicates a high ability of the model to distinguish high-risk areas from low-risk areas (Supplementary Fig. S2).

**Figure 6:**
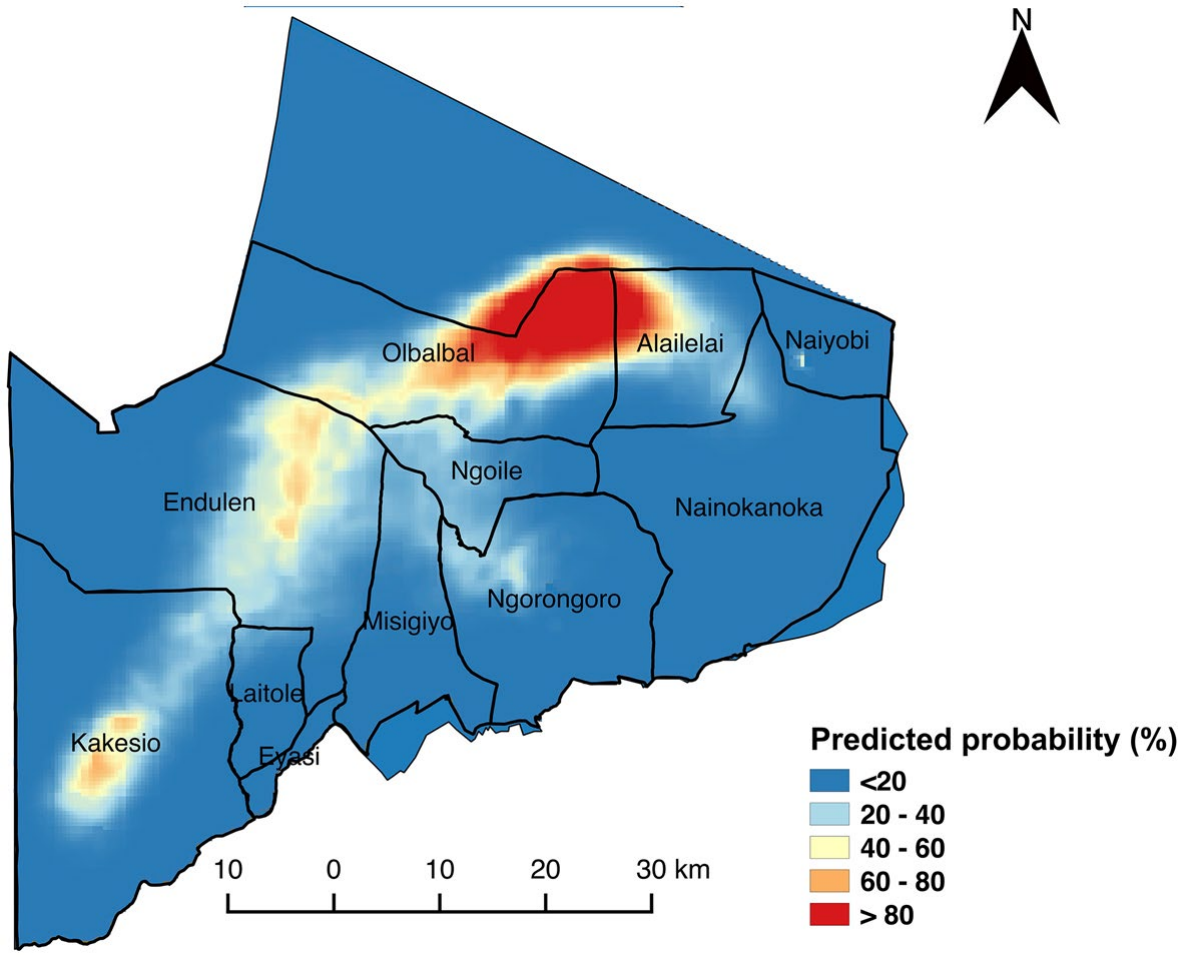
Predicted probability of being an anthrax-risk area in the Ngorongoro Conservation Area. The predicted probability of risk is based on data defining high-risk areas, generated through participatory mapping, and explained by environmental variables associated with the risk of anthrax. The map was created in QGIS 2.18.2; the Ngorongoro Conservation Area was sourced from the National Bureau of Statistics, Tanzania (http://www.nbs.go.tz/).

The level of risk predicted by the model indicated that areas with the greatest risks of anthrax in the NCA are in Olbalbal ward extending into Alailelai, Endulen, Kakesio and Ngoile wards (Fig. 6).

## Discussion

The occurrence of endemic diseases such as anthrax in rural and remote low-resource settings necessitates practical solutions for prioritization and targeted control. This study demonstrates that participatory mapping approaches reliant on indigenous knowledge of disease and the environment have the potential to be used as a surveillance tool for anthrax in areas where traditional methods may be challenging to implement due to infrastructural and financial constraints, as well as animal management practices. We applied GIS technologies to environmental data and data on anthrax risk gathered through participatory mapping in an endemic setting of rural Africa. Our analyses enhanced our understanding of the underlying environmental factors associated with areas of perceived risk, for example proximity to water bodies and low organic carbon content. We were also able to make predictions regarding the spatial distribution of risk. These findings suggest that in the NCA, factors that enable contact with *B. anthracis* spores are more important drivers of anthrax risk, compared to those that support spore survival.

Participatory mapping, which has long been applied to aid decision making for resource management, can also support epidemiological studies^11,12,25^ as it captures data that are difficult to obtain using standard methods^38^ because of underreporting. In addition, in the case of anthrax, locations of cases or sampling may not represent actual risk areas, due to the nomadic lifestyle of affected communities. In line with other studies, here we show that maps produced through participatory methods can help identify priority areas for intervention^12,39,40^. The additional application of GIS and statistical methodology^25^ provides further opportunities to study environmental conditions that influence disease occurrence, similarly to research on malaria vector control^13^. Here we combine these methods to improve our understanding of anthrax risk areas and environmental drivers of risk as an alternative to incidence data^2,20,22^.

The association between proximity to water bodies and high anthrax risk suggested by initial data exploration that ignored spatial autocorrelation gave potentially misleading results—high-risk areas were farther from water bodies compared to low-risk areas (Fig. 4). In contrast, statistical analyses that accounted for this autocorrelation showed that perceived high-risk areas were closer to water sources, a finding consistent with previous studies^20,22^. Such risk is likely driven by contamination with carcasses of infected animals seeking water during the late stages of the disease^14^ that contributes to spore accumulation. Dry periods typified by the congregation of large numbers of animals at watering points pose additional risks. Anthrax outbreaks occurring close to water sources have indeed been documented extensively^20,22,41,42^.

Previous studies have found soils with high organic content to be associated with anthrax spore persistence^19,43,44^. SOC is usually determined by a combination of organic matter from plant and animal tissue residues and microbial biomass^45^. Organic matter is thought to promote anthrax risk in two ways. Firstly, it favours cycles of germination and sporulation—the disputed “incubator area” hypothesis put forward by Van Ness^43,46^. Secondly, it may attract and sequester spores, shielding them from environmental damage^19^, creating a high concentration of spores (at sufficient infectious doses) in particular locations. However, in our study, we found high risk of anthrax to be associated with lower soil organic matter. Since plant biomass is a major contributor to SOC^45^, it is likely to be related to vegetation index, and low SOC might mean low vegetation in high-risk areas as observed in our univariable analysis with EVI (Table 3). The relationship between SOC and anthrax risk may suggest a meaningful association with EVI, which was removed from the multivariable analysis for statistical reasons. In areas where vegetation height is low, animals may ingest soil with spores more easily^47^, and this could potentially explain our results. Taken together with proximity to water bodies, this suggests that in the NCA, and possibly ecologically similar areas in sub-Saharan Africa, the risk of anthrax is associated with factors that promote host contact with *B. anthracis* spores rather than spore survival.

While CEC, pH and slope were not clearly associated with the probability of points falling in high-risk areas, based on previous studies, these factors are likely to contribute to anthrax risk in certain environments by facilitating spore persistence. Higher soil calcium levels and alkaline pH have been previously associated with high-risk areas in larger geographical locations in Tanzania^20,48^ and elsewhere^43,44,49^. Steenkamp et al*.* found no association of anthrax occurrence with pH in the Kruger National Park^22^. With regards to slope, anthrax has been found to occur more frequently in steppe areas^19^ which are characterised by large arid and flat grasslands. There is a possibility that run-off on higher slope may help to concentrate spores in water holes or depressions, which provides an additional explanation for increased anthrax risk closer to water bodies. The lack of non-ambiguous support for these particular factors in this study may be due to (1) the use of CEC as a proxy for calcium; (2) the small spatial scale of the study (i.e. focusing on the NCA only) which may have led to limited variation in the environmental conditions across the area; and/or (3) variability in the environmental conditions and patterns associated with anthrax occurrence in endemic versus sporadic outbreak situations or long-term versus short-term survival of spores, both of which are poorly understood. It could be hypothesized that the environmental conditions in a locality may select for *B. anthracis* spores that survive and persist in such conditions, if facilitated by other risk factors that promote high occurrence and sustain endemicity. A long history of anthrax occurrence might indicate that *B. anthracis* can survive in local environmental conditions that differ from those that are putatively associated with the pathogen (or disease). Spore persistence may therefore mask environmental effects if these change over time.

Varying climatic conditions may prevail in different locations affected by the same disease. For instance, a previous study suggested diverse ecological factors may be associated with anthrax risk, presenting a challenge in predicting the risk of disease in new locations using models of previously known high-risk areas^50^. In northern Tanzania, the study by Hampson et al*.*^20^, found that anthrax can be frequently observed in a number of habitats. Across the NCA, a diversity of climatic conditions can be observed at a given timepoint^51^. This may make it difficult to generalise findings across different locations and may explain why in our study some of the variables were not statistically different between high- and low-risk areas. Historically (before the mid-twentieth century), anthrax was observed frequently (and is still sporadically reported) in temperate^52^ and boreal regions such as in Scandinavia, Russia and North America^53,54^, areas with ecological characteristics distinct from those in tropical Africa. It is therefore plausible that *B. anthracis* spores evolved to survive in a wide range of environments and that local conditions that facilitate transmission to suitable hosts are more important than factors affecting spore survival.

The AUC of 94% suggests that the fitted model accurately predicts high- and low-risk areas. However, we caution that impressive predictive accuracy based on within-sample prediction can result from overfitting (inclusion of spurious predictors in the model), especially where spatial autocorrelation is strong. We guarded against this source of overfitting by accounting for spatial autocorrelation in the regression model, and we therefore have confidence in the significant associations with DOWS and SOC. We explored using out-of-sample prediction to estimate AUC by fitting the model after leaving out 25% of the study area, but found that model fitting was unstable when a reduced study area was used. Strong validation of the predictive accuracy of the model would therefore require new data from a similar area, or from an extension of this study to a larger study area with more independent risk areas. Further data currently being collected regionally promise to provide such opportunities.

The data obtained using participatory mapping provided descriptive as well as inferential information about the pattern of environmental factors present in high-risk areas. However, this type of data reliant on local knowledge may be limited by subjectivity. Our participatory mapping approach could be refined and improved. For example, conducting participatory mapping a number of times and combining the resulting data would reduce the effect of subjectivity and would enable a better understanding of the uncertainty to be expected. Another improvement we propose relates to the use of other criteria to exclude locations not accessed by livestock. While an alternative criterion such as elevation could be used, it is difficult to define a threshold which excludes animals. In addition, the strategy may not take into account other criteria, for example inaccessible forested areas at ‘accessible’ elevations, or areas with accessible elevation but made inaccessible by surrounding areas. Overall, because the data on settlements we used to exclude inaccessible locations comprised temporary settlements set up in the dry season after long distance movements, we expect that areas most likely to pose a risk to livestock have been identified.

Overall, our approach can help guide the selection of geographical locations and strategies for prioritisation of anthrax control. This is of particular importance in settings where the disease burden is high, but resources to address the problem are limited. However, strategies would need to be tailored to the specific needs of the communities residing in this and similar areas. For example, we show that high-risk areas for anthrax occupy central parts of the NCA where key livestock resource areas such as water holes are located. Depending on the direction of movements, this makes it challenging to traverse the NCA without encountering a high-risk area, with particular implications for seasonal north–south movements in search for pasture and water, characteristic of the pastoral livestock management system in the NCA. Animal movement restriction is often recommended as an effective strategy for the control of anthrax^55,56^ and infectious diseases more generally. However, in most of Africa, livestock-keeping communities depend on movements to resource areas (particularly grazing and watering points) to ensure the survival of their animals and this would need to be taken into account in any management recommendations. Therefore, vaccination-based strategies prioritising high-risk areas and herds making use of these resources appear to be the most viable approaches for reducing the burden of anthrax in these communities.

## Conclusions

This study demonstrates the value of participatory mapping techniques for understanding disease distribution and identifying priority areas for control in low-resource areas that would ultimately benefit public health. GIS technologies are increasingly becoming available in developing countries and can be combined with participatory approaches to generate useful data. A combination of GIS tools and participatory approaches yielded information about risk areas and the environmental conditions associated with those areas in a setting hyper-endemic for anthrax. In our study, anthrax occurs mostly in areas characterised by low organic matter and in proximity to water bodies. This suggests that the transmission of *B. anthracis* to animals likely drives the risk of disease, more than factors that favour the survival of spores in the environment. Interventions for local anthrax control may thus be most effective if targeted towards at-risk areas and livestock management practices that limit transmission to animals. Our general approach may be of value to the surveillance and control of anthrax and other diseases in other contexts under similar constraints.

## Supplementary Information

Supplementary Information.

## Data Availability

The datasets generated and analysed during the study are available from the corresponding author on reasonable request.

## Abbreviations

AUC: Area under the curve
CEC: Cation exchange capacity
CI: Credible interval
CIESIN: Center for International Earth Science Information Network
COSTECH: Tanzania Commission for Science and Technology, Tanzania
DOWS: Distance to inland water bodies
EVI: Average enhanced vegetation index
GIS: Geographic Information System
LSTD: Average daytime land surface temperature
NBS: National Bureau of Statistics, Tanzania
NCA: Ngorongoro Conservation Area
NIMR: National Institute for Medical Research, Tanzania
OR: Odds ratio
pH: Measure of acidity or alkalinity
QGIS: OpenSource Geographic Information System
SD: Standard deviation
SOC: Predicted topsoil organic carbon content
UTM: Universal Transverse Mercator coordinate system
VIF: Variance inflation factor
