# Supplementary material for: Participatory mapping identifies risk areas and environmental predictors of endemic anthrax in rural Africa
Source: Sci Rep. 2022 Jun 22;12:10514. doi: 10.1038/s41598-022-14081-5 (PMC9217952; doi:10.1038/s41598-022-14081-5)
Supplement: Supplementary file 1 — Supplementary Information. [file 41598_2022_14081_MOESM1_ESM.pdf]

Additional File 1.

Supplementary methods

Participatory mapping

Participatory mapping sessions were held as part of 10 Focus Group Discussions (FGDs) that were attended by participants from all 11 administrative wards (the largest administrative subunits) in the study area – one exercise was held for two wards. Study participants included livestock field officers, community leaders and village or ward officers, all of whom owned livestock and were conversant with livestock management and health matters in the area.

In order to assess the extent of community-level understanding of anthrax in the area, we hosted a series of workshops before initiation of research. In addition, the FGDs involving participatory mapping sessions were used to understand local knowledge of anthrax in humans and animals as well as practices, locations and periods of the year that pose particular risks. Finally, the consenting process conducted prior to data collection included a brief description of the disease and how it is transmitted. While we do not present these results here, these data, as well as data collected during randomised household surveys (n=209) in the area, showed that there is a high level of anthrax awareness and ability of communities to identify infection signs and sources in both animals and humans [1]. We also identified key practices around carcass consumption and handling, and management of livestock and their products that explain high exposure rates, particularly in some locations and periods of the year. Further research we have conducted in the area based on diagnostic confirmation of cases [2] demonstrates high case recognition ability by livestock owners: over 73% (403 out of 548) of animal carcasses reported by community members and tested by molecular methods were positive, with a further 11 samples that gave suspect results. While we did not assess systematically whether individual participants in FGDs had experienced anthrax in their herds or households, in the 209 surveys mentioned above one third of households reported having lost livestock to anthrax at some point in the past [1]. FGD participants also described the disease as having a strong presence in the area and that livestock deaths due to the disease are commonplace. On this basis, we are confident that communities in this setting have a clear understanding of the disease, are able to recognise suspect cases and suffer frequent losses due to the disease throughout the year with the highest burden during the

dry season. For example, the local term used for animal anthrax literally means “hot disease” due to its greater occurrence during the “dry and hot season”.

Earlier studies we conducted to investigate ecological and (animal) behavioural drivers of risk, highlighted spatial heterogeneity in anthrax cases and seroprevalence [3,4]. This earlier work provided the foundation for validating the ability of communities to identify high-risk areas. Specifically, during participatory mapping sessions participants were asked to identify geographical locations on maps they perceived as areas where their animals could be exposed to anthrax (as opposed to areas where they succumbed to disease). However, we also asked for generic information on locations where anthrax outbreaks had occurred in the past and that could be targeted for active investigation of cases [1]. We focused on areas where animals had been taken to prior to disease rather than where they had died because movements to contaminated pasture, water, soil or mineral licks are considered as important factors of risk. While we did not assess frequency of use of these areas as part of the FGDs, participants reported that these are key resource areas accessed for grazing and watering, and that choice of areas is strictly season-dependent. While pasture and water are generally readily available during the wet season, minerals are not, hence movements in search for mineral salts occur more regularly independently of the season.

A typical resilience mechanism against anthrax is the movement away from possible sources of infection, for example to a different location considered to be free of the disease. It is therefore unlikely that participants would be reluctant to identify locations close to their households as risk areas.

Supplementary results

A) Multicollinearity of variables

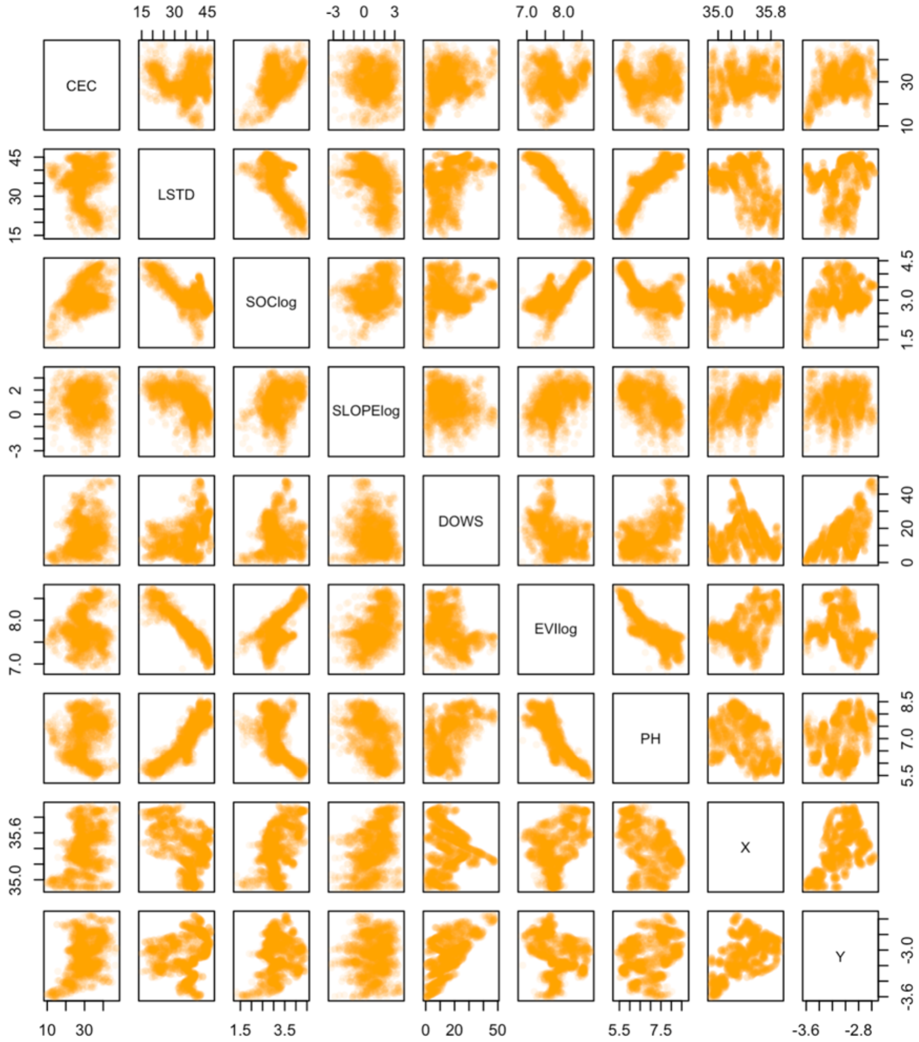

Supp Fig S1. Scatterplot of variables - cation exchange capacity (CEC), average daytime land surface temperature (LSTD), predicted topsoil organic carbon content (SOC), slope, distance to inland water bodies (DOWS), average enhanced vegetation index (EVI), and pH - showing evidence of collinearity. Variance inflation factor was used to exclude LSTD and EVI from multivariable analysis.

B) Accuracy of the multivariable model

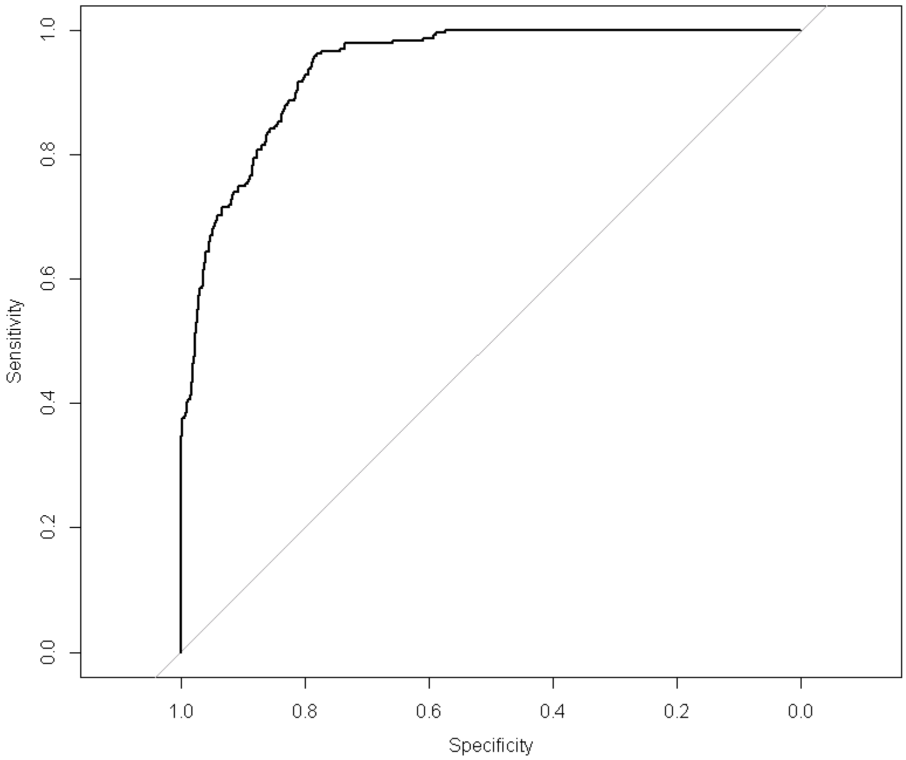

Supp Fig S2. Receiver operating characteristic (ROC) curve obtained to assess model performance. The area under the curve (AUC) was 0.94, indicating a good within-sample predictive performance.

C) Results of univariable and multivariable generalized linear mixed models.

Results of Generalized Linear Mixed Models with Robust Random Fields for Spatiotemporal Modeling (glmmfields), investigating the effect of predictor variables on the probability of points falling into high anthrax risk areas. Mean, standard error (se), standard deviation (sd), quantiles of posterior distribution and effective sample size (n_eff) are shown. CEC = Cation exchange capacity, DOWS = Distance to inland water bodies, EVI = Average enhanced vegetation index, LSTD = Average daytime temperature, SOC = Predicted topsoil organic carbon content.

Supp Table S1. Univariable models.

Model	mean	se	sd	2.5%	25%	50%	75%	97.5%	n_eff	Rhat
CEC	0.02	0.00	0.02	-0.07	-0.04	-0.02	0.00	0.02	1780	1.00
pH	0.92	0.01	0.29	0.34	0.73	0.92	1.11	1.52	3058	1
DOWS	-0.13	0.00	0.04	-0.21	-0.16	-0.13	-0.10	-0.05	1356	1.00
log(EVI)	-3.82	0.01	0.68	-5.17	-4.28	-3.80	-3.35	-2.52	2193	1.00
log(LSTD)	8.88	0.03	1.65	5.76	7.77	8.83	9.97	12.21	2737	1.00
log(Slope)	0.22	0.00	0.12	-0.01	0.14	0.22	0.30	0.45	2972	1.00
SOC	-2.80	0.01	0.50	-3.82	-3.13	-2.79	-2.45	-1.86	2466	1.00

Supp Table S2. Multivariable model

Model	mean	se	sd	2.5%	25%	50%	75%	97.5%	n_eff	Rhat
CEC	0.002	2.00×10^{-3}	2.00×10^{-3}	2.00×10^{-3}	2.00×10^{-3}	2.00×10^{-3}	2.00×10^{-3}	2.00×10^{-3}	3133	1.00
pH	5.27×10^{-1}	5.27×10^{-1}	5.27×10^{-1}	5.27×10^{-1}	5.27×10^{-1}	5.27×10^{-1}	5.27×10^{-1}	5.27×10^{-1}	4861	1.00
log(SOC)	-2.55	-2.55	-2.55	-2.55	-2.55	-2.55	-2.55	-2.55	5289	1.00
log(slope)	3.60×10^{-1}	$3.60 \times 10_{-1}$	3.60×10^{-1}	3.60×10^{-1}	3.60×10^{-1}	3.60×10^{-1}	3.60×10^{-1}	3.60×10^{-1}	6637	1.00
DOWS	-9.73×10^{-2}	-9.73×10^{-2}	-9.73×10^{-2}	-9.73×10^{-2}	-9.73×10^{-2}	-9.73×10^{-2}	-9.73×10^{-2}	-9.73×10^{-2}	3295	1.00

EVI and LSTD were removed from the multivariable model due to collinearity with other variables as indicated by a variance inflation factor greater than 3 [5].

Supplementary References

1. Aminu OR (2020). Enhancing surveillance and quantifying impacts to improve our understanding of endemic anthrax in low resource settings. PhD thesis, University of Glasgow.
2. Aminu OR, Lembo T, Zadoks RN, Biek R, Lewis S, Kiwelu I, Mmbaga BT, Mshanga D, Shirima G, Denwood M and Forde T (2020). Practical and effective diagnosis of animal anthrax in endemic low-resource settings. PLoS Negl Trop Dis 14(9): e0008655.
3. Hampson K, Lembo T, Halliday J, Auty H, Packer C, Bessell P, Fyumagwa R, Hoare R, Beesley CA, Stamey K, Ernest E, Mentzel C, Mlengeya T, Roberts K, Wilkins PP and Cleaveland S (2011). Predictability of anthrax outbreaks in the Serengeti, Tanzania. J Appl Ecol 48 (6):1333-1344.
4. Lembo T, Hampson K, Auty H, Beesley CA, Bessell P, Packer C, Halliday J, Fyumagwa R, Hoare R, Ernest E, Mentzel C, Mlengeya T, Stamey K, Wilkins PP and Cleaveland S (2011). Serologic surveillance of anthrax in the Serengeti Ecosystem, Tanzania, 1996–2009. Emerg Infect Dis 17(3):387–394.
5. Hair JF, Black WC, Babin BJ, Anderson RE. Multivariate Data Analysis Joseph F. Hair Jr . William C . Seventh Ed. Black; 2010. 733 p.
